# Relationship between intraperitoneal volume and intraperitoneal pressure during peritoneal dialysis—a pilot study in adult patients

**DOI:** 10.14814/phy2.70179

**Published:** 2025-03-12

**Authors:** Fansan Zhu, Laura Rosales Merlo, Lela Tisdale, Maricar Villarama, Jun Yi, Zahin Haq, Xiaoling Wang, Nadja Grobe, Karsten Fischer, Kulwinder Plahey, Richard A. Lasher, Paul Chamney, Brigitte Schiller, Peter Kotanko

**Affiliations:** ^1^ Renal Research Institute New York City New York USA; ^2^ Mount Sinai Hospital New York City New York USA; ^3^ Fresenius Medical Care North America Waltham Massachusetts USA; ^4^ Fresenius Medical Care AG Bad Homburg Germany; ^5^ Icahn School of Medicine at Mount Sinai New York City New York USA

**Keywords:** bioimpedance, intraperitoneal pressure, intraperitoneal volume, peritoneal dialysis, ultrafiltration

## Abstract

Monitoring intraperitoneal pressure (IPP) offers valuable insights into changes of intraperitoneal volume (IPV) during peritoneal dialysis (PD). This study aims to investigate the relationship between IPV and IPP during a PD dwell. Thirteen patients were studied during a 2‐h dwell using 2 L of dialysate containing 2.5% dextrose. IPP was measured using a pressure sensor integrated into an automated PD cycler. IPV was monitored concurrently by segmental bioimpedance (Hydra 4200). The density (*ρ*) of the PD dialysate was measured using a meter, and the creatinine and glucose concentrations in both dialysate (D) and serum (P) were measured pre‐ and post‐PD dwell. A physical model (IPP = *ρ* × *g* × *h*), was used to describe the relationship between IPP and IPV, where h is the apparent dialysate height and g is the gravitational acceleration. The change in IPP (ΔIPP, −21.2 ± 18%) was mainly determined by the change of h (Δh, −20.9 ± 18.5%), while the change *ρ* (Δ*ρ*, −0.34 ± 0.06%), was minor. The study demonstrated an association between ΔIPP and the ratio of D/P creatinine and D/D_0_ glucose, suggesting that ΔIPP may reflect membrane transport characteristics. Due to its noninvasive and seamless nature, the clinical utility of PD cycler‐based measurement of IPP warrants further exploration.

## 1 INTRODUCTION

Understanding of peritoneal membrane characteristics is pivotal for the success of peritoneal dialysis (PD). To this end, various tests, such as the peritoneal equilibration test (PET), are routinely employed in clinical practice. Regrettably, the PET is cumbersome and conducted infrequently in clinical practice; while the measurement of intraperitoneal volume (IPV) during the dwell offers a more direct method for observing fluid transport across the peritoneal membrane (Flessner, 1992; Morelle et al., 2021; Twardowski et al., 1983). Presently, a volume marker, such as ^125^I‐labeled albumin, is used to measure IPV during a PD dwell (Asghar & Davies, 2008; Heimburger et al., 1992; Stachowska‐Pietka et al., 2019). However, it is worth noting that this dilution method cannot be readily applied in routine clinical practice, as it requires a radioactive tracer (Teixido‐Planas et al., 2014). The three‐pore modeling method holds utility not only in clinical applications but also in theoretical contexts to elucidate the transfer of toxins and fluid between the peritoneal membrane (Asghar et al., 2005; Devuyst & Rippe, 2014; Lindholm & Bergstrom, 1992). Previous studies have documented the relationship between IPV and intraperitoneal pressure (IPP) (Li et al., 2023; Perez‐Diaz et al., 2021). While changes in IPP may indicate fluctuations in IPV during the dwell (Schneditz et al., 2020), it is noteworthy that studies investigating the relationship between IPP and IPV have yielded conflicting results (Durand et al., 1993; Fischbach et al., 2003; Perez Diaz et al., 2017; Twardowski et al., 1983, 1986). On one hand, there is evidence suggesting a positive correlation between IPP and IPV, particularly with the instillation of large volumes (more than 2 L) of dialysate into the peritoneal cavity (Dejardin et al., 2007; Twardowski et al., 1986, 1983). Conversely, there are findings indicating an inverse correlation between IPP and the change in IPV during the dwell period (Durand et al., 1993; Perez‐Diaz et al., 2021). This confusion may, in part, stem from the current measurement methods, which typically involve manual and intermittent measurements of IPP (Durand et al., 1992; Sigogne et al., 2020). Exactly, IPP measurements might not capture the pressure dynamics with the necessary temporal resolution. In our previous study, we employed abdominal segmental bioimpedance analysis (SBIA) to track fluid changes in the peritoneal cavity (Zhu et al., 2024). Through this method, we established a correlation between the maximal ultrafiltration volume (UFV) and the transport characteristics of the peritoneal membrane as assessed by the PET (Zhu et al., 2019). Certainly, abdominal SBIA necessitates the utilization of a specific device, such as the Hydra 4200, along with a switch, electrodes, software, and a trained operator to perform the measurements accurately (Zhu et al., 2003). A recent publication has confirmed that SBIA is a useful method to monitor change in IPV in PD patients (Liu et al., 2023).

In this study, we explore a quasi‐continuous measurement of IPP utilizing a pressure sensor integrated into a commercially available automated PD cycler (Fresenius Medical Care, Waltham, MA, USA). Furthermore, we put forward a physical model aiming to elucidate the interplay among IPV, IPP, and dialysate density.

The objectives of this study were: (1) Assessing the accuracy of the pressure sensor integrated into the automated PD cycler through a bench study. (2) Exploring the connection between alterations in IPP and IPV in PD patients throughout the dwell period. and (3) Evaluating the potential association between IPP and membrane transport characteristics.

## 2 METHODS

### 2.1 Physical model of dialysate pressure and volume in the peritoneal cavity

**FIGURE 1:**
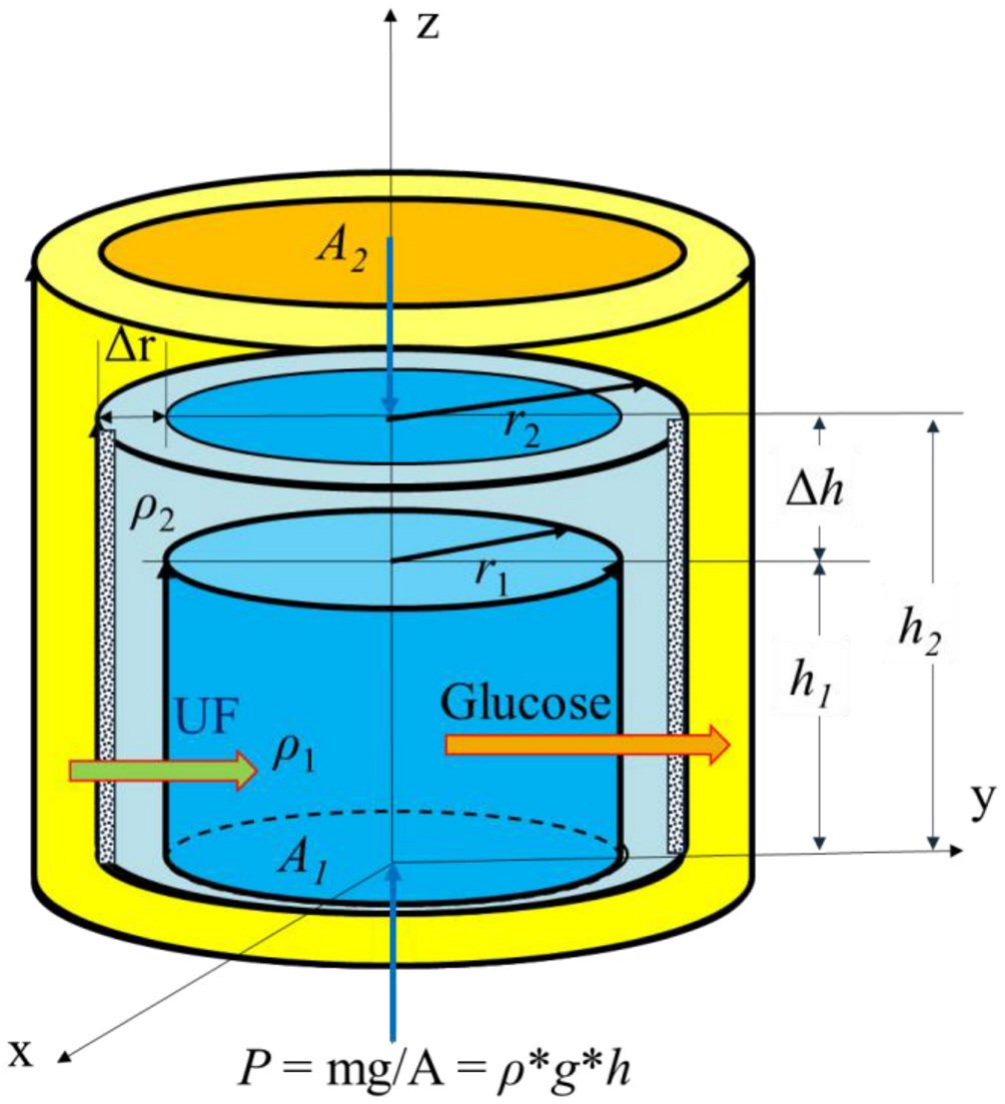
A model of description of the relationship between intraperitoneal pressure (IPP) and intraperitoneal volume (IPV) with change in dialysate height (Δ*h* = *h*_*2*_−*h*_*1*_) and change in density (Δ*ρ* = *ρ*_*2*_−*ρ*_*1*_). Parameters *r*_*1*_ and *r*_*2*_ represent the radius of peritoneal cavity in the beginning and it reached the maximal radius (Δ*r* = *r*_*2*_−*r*_*1*_). A is the cross‐section area which can be calculated by IPV/*h*. The increase in the *A* (Δ*A* = *A*_*2*_−*A*_*1*_) is due to fluid transferring from excess fluid in the surrounding tissue and in internal organs.

The peritoneal cavity is conceptualized as a closed container with uniform pressure distribution and a constant temperature. Figure 1 illustrates the idealized physical model depicting the relationship between fluid height (h), cross‐sectional area (A), and density (*ρ*) of dialysate in the peritoneal cavity. IPP is defined as the vertical downward pressure. In this schematic representation, the inner blue area represents the fresh dialysate with density *ρ*_1_. The middle light blue area represents the fluid volume with density *ρ*_2_, where volume increases due to ultrafiltration (UF) and density changes occur due to factors such as glucose absorption (AP). The outer yellow area represents the tissue external to the peritoneal membrane. Certainly, due to the elastic properties of the peritoneal cavity, an increase in IPV can result in the expansion of the cavity, causing the radius to increase from *r*_1_ to *r*_2_ and lead to increase the cross‐sectional area from *A*_1_ to *A*_2_ of the cavity. Indeed, once the cross‐sectional area (*A*) of the peritoneal cavity reaches its maximum (*A*_2_), the radius will remain constant. This indicates a limit to the expansion capacity of the peritoneal cavity, governed by its elastic properties. If IPV continues to increase after the cross‐sectional area has reached its maximum, the height of the dialysate in the peritoneal cavity will correspondingly increase from *h*_1_ to *h*_2_. Consequently, the relationship between the change in height (Δ*h*) and the change in cross‐sectional area (Δ*A*) is determined by factors such as ultrafiltration volume (UFV) and subject‐specific membrane transport characteristics.

As the sum of pressures in the surrounding area along the *x* and *y* axes equals to zero, the pressure of fluid in the peritoneal cavity is solely considered in the vertical direction (*z* axis) due to gravity, as illustrated in Figure 1. The basic equation describing pressure (*P*) and its related factors is as follows:

(1) P=M×g/A

where *M* is the mass (kg) of fluid in peritoneal cavity, *g* (m/s^2^) is the acceleration of gravity, and *A* (m^2^) is the cross‐sectional area of the fluid in the peritoneal cavity.

Since *M* × *g* / *A* = *g* × *h* × *M* /(*A* × *h*) = *g* × *h* × *M* / *V*, the pressure can be presented as

(2) P=ρ×g×h

where *P* represents intraperitoneal pressure (IPP, kg/(m*s^2^)), *ρ* (*M/V*, kg/m^3^) is the density of dialysate in peritoneal cavity, *g* (m/s^2^) is the acceleration of gravity, and *h* (m) is the height of the fluid in the peritoneal cavity.

It's important to note that the *ρ* of the dialysate may change during the dwell period due to alterations in the dialysate composition. For instance, as water enters the peritoneal cavity via ultrafiltration (UF) and glucose moves to body tissue through reabsorption, the density of the dialysate decreases over time. Hence, the change in intraperitoneal pressure (ΔIPP) is inversely associated with the change in density (Δ*ρ*):

(3) ΔIPP=−a×∣Δρ∣+b×

where *a* is a constant factor for individual patients and *b* serves as the intercept. Δ*ρ* is the difference between post‐ and pre‐PD, and post *ρ* ≤ pre *ρ*, we can achieve Δ*IPP* ≤0 if the height of dialysate remains constant or decreases (Δ*h* ≤ 0).

According to Equation 2, once *ρ* and IPP are determined, the apparent height of dialysate can be calculated using the following equation:

(4) h=ρ×g/IPP

Alternatively, if we assume the density is constant, the change in IPP can be calculated by the change in the height of dialysate, as shown in the following equation:

(5) ΔIPP=g×ρ×Δh

It must be noted that this relationship (Equation 5) is correct only when the change in the density is smaller than change in height of dialysate. Since Δ*h* = Δ*IPV/A*, the relationship between ΔIPP and ΔIPV (UFV) can be deduced as Equation 6:

(6) $$\Delta \mathrm{IPP}=\frac{\rho \times g}{A}\Delta \mathrm{IPV}$$

where A is the apparent cross‐sectional area and A = IPV/h. An important assumption for Equation 6 is that the height of dialysate in peritoneal cavity will increase when the density and cross‐sectional area are constant or subjected to small changes.

However, if the height (h) and density (*ρ*) both are variables, we need to ascertain the relationship of pressure to two variables. From Equation 2, we obtained partial derivative equation shown below:

(7) $$\frac{\partial \mathrm{IPP}}{\partial t}=g\times h\times \frac{\partial \rho }{\partial t}+\rho \times g\times \frac{\partial h}{\partial t}$$

where $\frac{\partial \mathrm{IPP}}{\partial t}$ represents IPP change in function of time, $\frac{\partial \rho }{\partial t}$ and $\frac{\partial h}{\partial t}$ represents change in the density and height of the dialysate solution during PD treatment respectively. We assume that the change in density (Δ*ρ* = post *ρ* – pre *ρ*) of dialysate is ≤0 because the post dialysate density usually decreases when ultrafiltration volume (UFV) increases and with absorption of glucose during dwell. Since Δ*ρ* ≤ 0, change IPP can be presented as follows:

(8) $$\Delta \mathrm{IPP}=g\left[\rho \times \mathrm{\Delta h}-h\times \Delta \rho \right]$$

Eq. 8 shows that direction (>0 or <0) of Δ*IPP* is determined by ρ×Δh−h×Δρ. Since the value of Δ*ρ* is smaller than Δ*h*, Δ*h* is main component to domain the Δ*IPP*. According to the relationship between volume (*V*) and height (*h*) in a standard cylinder (Figure 1), we have a simple equation below.

(9) IPV=h×A

where *h* and *A* are apparent height and cross‐sectional area of dialysate in the peritoneal cavity. If *h* and *A* both are variables, we can use partial derivative to obtain the equation as follows.

(10) $$\Delta h=\frac{1}{A_{1}}\left[\Delta \mathrm{IPV}-h_{1}\times \Delta A\right]$$

where *A*_*1*_ and *h*_*1*_ are initial cross‐sectional area and height of dialysate in the peritoneal cavity respectively. Indeed, if the change in intraperitoneal volume (ΔIPV) exceeds *h*_1_ × Δ*A*, the change in height (Δ*h*) will increase from *h*_1_ to *h*_2_, leading to a positive change in ΔIPP where *h*_1_ is the initial height of the dialysate and Δ*A* is the change in cross‐sectional area of the peritoneal cavity. This scenario occurs when the elasticity of the peritoneal membrane allows for further expansion of the peritoneal cavity beyond its initial capacity.

Considering all conditions, the change in IPP can be calculated using the following equation:

(11) $$∆\mathrm{IPP}=\left\{\begin{array}{c} >0\;\text{ΔIPV}>h_{1}\times \Delta A\;\\ \;\\ \;\\ \leq 0\;\text{ΔIPV}\leq h_{1}\times \mathrm{\Delta A}\; \end{array}\right.$$

where ΔIPP, Δ*A* and ΔIPV denote the change in pressure, change in apparent cross‐sectional area, and change in IPV, respectively.

According to Eq. 8, the increase or decrease of IPP during the dwell phase is determined by changes in the height (Δ*h*) of the dialysate solution within the peritoneal cavity. Since both *h* and *A* related to change in IPV (ΔIPV), if ΔIPV > h_1_ × Δ*A*, ΔIPP will be a positive value; (2). However, if the ΔIPV ≤ h_1_*Δ*A*, height decreases or remains constant (Δh≤0), leading to a negative change in pressure. Therefore, Equation 11 can be defined as a threshold to determine the direction of the change in IPP.

### 2.2 Bench studies

**FIGURE 2:**
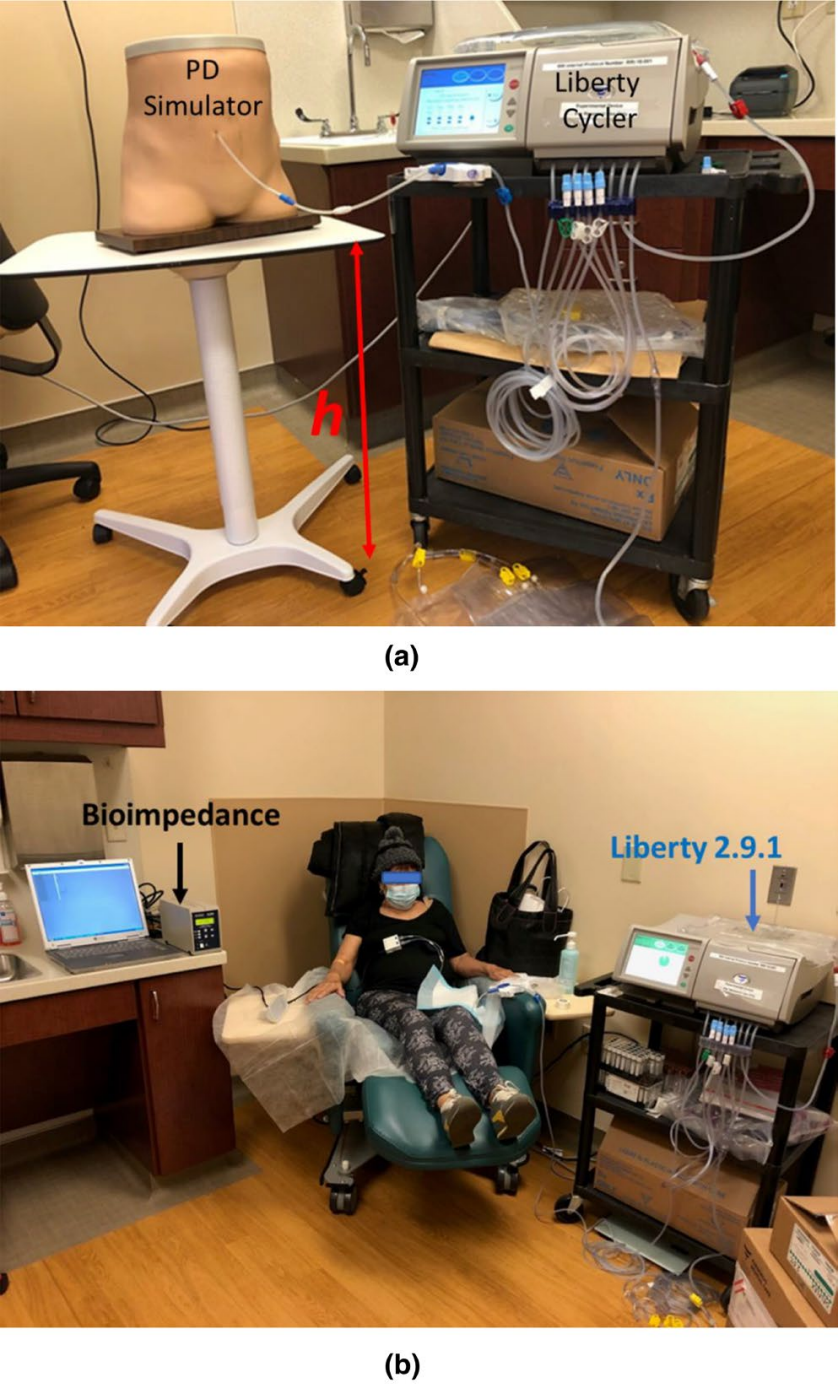
(a) Bench study for testing relationship of pressure with the height. (b) Devices set up for clinical study. Bioimpedance was for measurement of intraperitoneal volume and Liberty 2.9.1 was for monitoring intraperitoneal pressure.

To test the accuracy of the pressure sensor, we designed a bench study. This study involved comparing actual pressure changes, achieved by adjusting the height of a PD simulator, to the pressure measured from the sensor integrated into the automated PD cycler (Fresenius Medical Care, NA, Waltham, MA, USA). Figure 2 (a) illustrates the configuration of the bench study, which includes the automated PD cycler and a PD simulator (Life/form Peritoneal Dialysis Simulator, Fort Atkinson, WI USA) mounted on a height‐adjustable desk.

The pressure sensor records IPP data every 15 s. The PD simulator is constructed from a plastic container with a diameter of 150 cm and a height of 150 cm, filled with 2 liters of 2.5% dextrose dialysate. The height of the simulator gradually increased from the baseline (73 cm) in increments of 6.35 cm until reaching the highest point (91.4 cm). Afterward, the height is reduced back to the baseline in the same increments. The experiment consists of two phases of height changes: (a) Ascending from the baseline to the highest point. (b) Descending from the highest point back to the baseline. Linear regression analysis will be performed to compare the actual pressure with the sensor measurements.

To test the relationship between dialysate density and glucose concentration, the density of the fresh dialysate was measured as an average of three repeated measurements with standard PD solutions (1.5%, 2.5%, and 4.25%) and mixed solutions (1.0%, 2.0%, 3.0%, 3.5%, and 4.0%).

## 3 CLINICAL STUDY

### 3.1 Patients

We conducted a pilot study involving patients undergoing a 2‐h dwell with 2 L of 2.5% dextrose dialysate. The study received approval from the Western Institutional Review Board (WIRB Protocol #20202653). All participants provided written informed consent.

### 3.2 Measurements

The patients were seated in dialysis chairs with their legs positioned horizontally and at an approximate angle of 120° to the trunk during the peritoneal dialysis (PD) treatment (Figure 2b). The methodology for measuring IPV using SBIA has been detailed in previous publications (Zhu, & M LR, Tisdale L, Villarama M, and Kotanko P., 2022). Eight electrodes were placed on the right and left sides of the body to measure resistance at 5 kHz in the lower abdominal area using segmental bioimpedance analysis (Hydra 4200). Changes in IPV during the dwell can be estimated based on the known ratio of change in fluid to change in resistance during filling. The algorithm used to calculate IPV has been previously described (Zhu et al., 2003; Zhu, & M LR, Tisdale L, Villarama M, and Kotanko P., 2022). IPP was measured every 15 s during the dwell using the pressure sensors integrated into an automated PD cycler with software version 2.9.3. The IPP data were downloaded from the automated PD cycler after the PD treatment. The distances between the patient chair (56 cm) and the height of the desk (80 cm) of the automated PD cycler were kept constant. Measurements of body weight were conducted for all patients before and after PD treatment. The weights and volumes of dialysate for both fresh and drainage were measured using a digital scale and indicated by the automated PD cycler, respectively. Additionally, the ultrafiltration volume (UFV_Cycler_) was provided by the automated PD cycler. Dialysate density was measured using a handheld density meter (Mettler‐Toledo, Columbus, OH, USA) at room temperature. It was assumed that the density of fresh dialysate could be considered constant for a particular glucose concentration (2.5%) in individual patients. The change in density (Δ*ρ* = post density‐ pre density) of dialysate during the dwell can be calculated by subtracting the predensity of the fresh 2.5% dextrose dialysate from the postdensity measured in the drained volume.

### 3.3 Laboratory chemical data collection

Dialysate and blood samples (10 mL) were collected both pre and post‐PD. The concentration of glucose, BUN, Creatinine, Na+, K+, Calcium and Urea in dialysate and serum samples were measured by Horiba Pentra C400 chemistry analyzer.

### 3.4 Data analysis

The data are reported with mean ± standard deviation. A difference was considered significant if the *p*‐value was less than 0.05. UFV_Cycler_ was utilized for the comparison of IPP data, as these values are determined by the automated PD cycler. IPP_Start_ and IPP_End_ were defined as the average of five measurements taken after the start of dwell and the average of five measurements taken just before the end of dwell, respectively. IPV data was collected every 10 min to facilitate the comparison of IPP data during the dwell period. Relative change in IPP, density and height of the dialysate fluid were calculated using the formula (Postvalue−Prevalue) × 100/Prevalue [%]. Linear regression analysis and Bland–Altman plot were used to assess the relationship and agreement between the two parameters. All statistical analyses were conducted using Prism 9 (GraphPad Software Inc., San Diego CA, USA).

## 4 RESULTS

### 4.1 Bench study results

**FIGURE 3:**
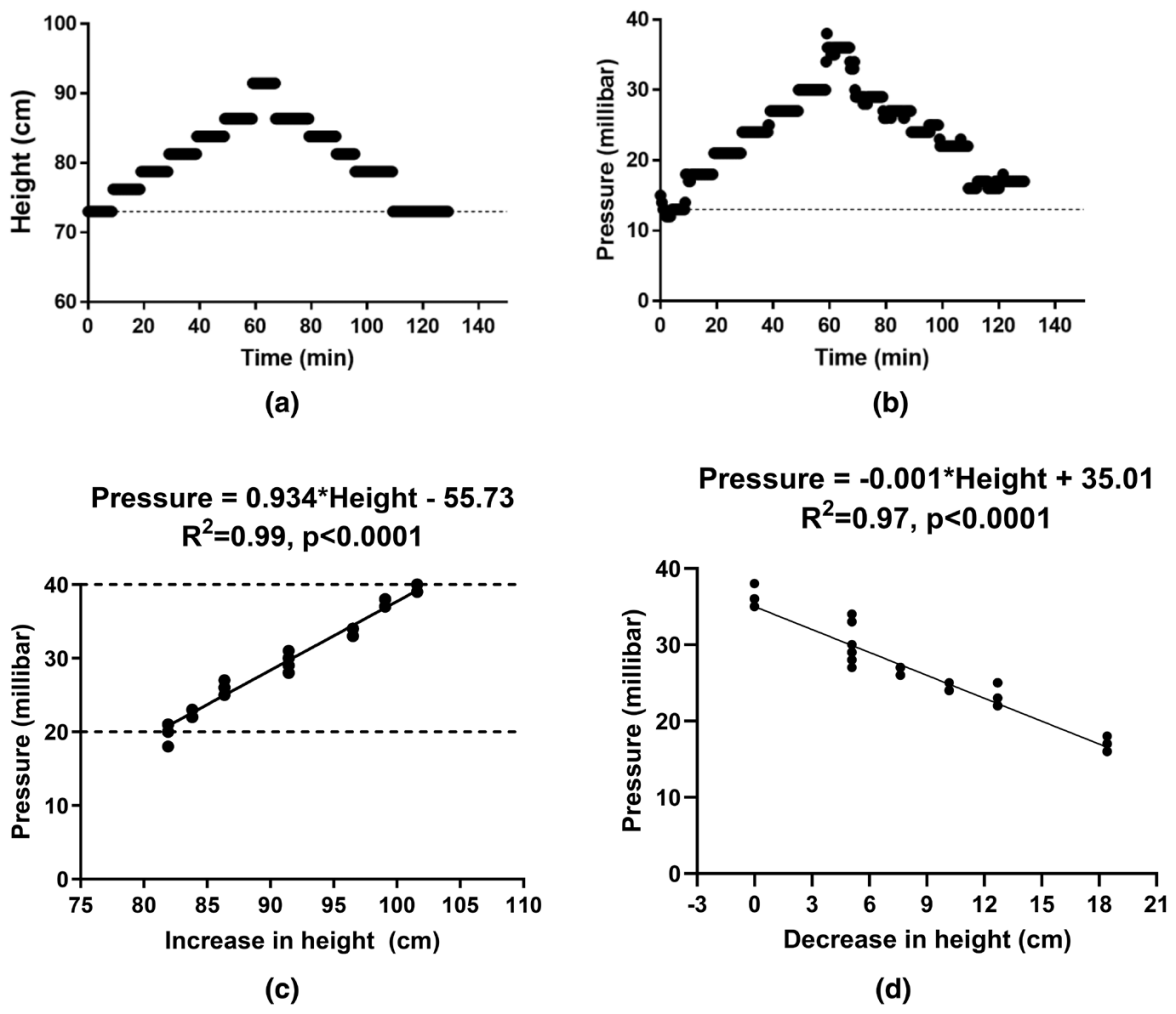
The bench study to test the relationship between height and IPP. (a) change in height of the simulator with time, (b) change in the pressure measured by the sensor integrated in Liberty cycler, (c) increased the pressure by increase in height of simulator (d) decrease in pressure by decrease height (Maximal height–decrease height) of the simulator.

**FIGURE 4:**
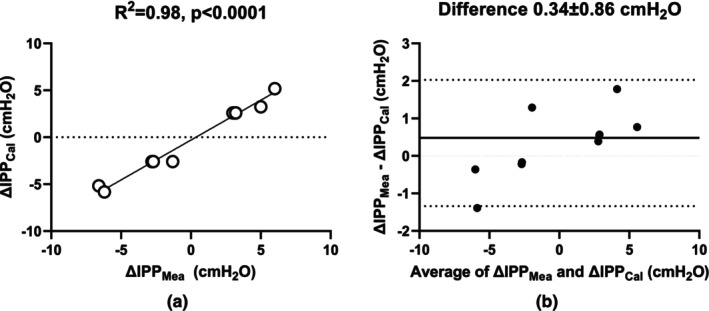
(a) Relationship between calculation of change in the pressure (ΔIPP = *ρ* × *g* × Δ*h*) by change in the height in each step and measurement of IPP with the sensor of Liberty Cycler, (b) Bland–Altman analysis with the data from (a).

The bench study revealed a strong relationship between the change in the height of the PD simulator (Figure 3a) and the corresponding change in pressure as measured by the sensor (Figure 3b). Pressure measurements (in millibar) were found to be linearly correlated with changes in the height of the PD simulator during both increases (*R* ^2^ = 0.99, Figure 3c) and decreases (*R* ^2^ = 0.97, Figure 3d). Furthermore, change in the pressure (ΔIPP_Cal_) can be calculated with the changing height (Δh) in each step of increase and decrease in the simulator according to equation (5). ΔIPP_Cal_ was highly correlated (*R* ^2^ = 0.98, *p* < 0.0001) with the measurement of IPP (ΔIPP_Mea_) by the sensor during the same timeframe (Figure 4a). Bland–Altman analysis revealed a small bias (0.34 ± 0.86 cmH_2_O) of IPP between measured and calculated IPP values (Figure 4b).

**FIGURE 5:**
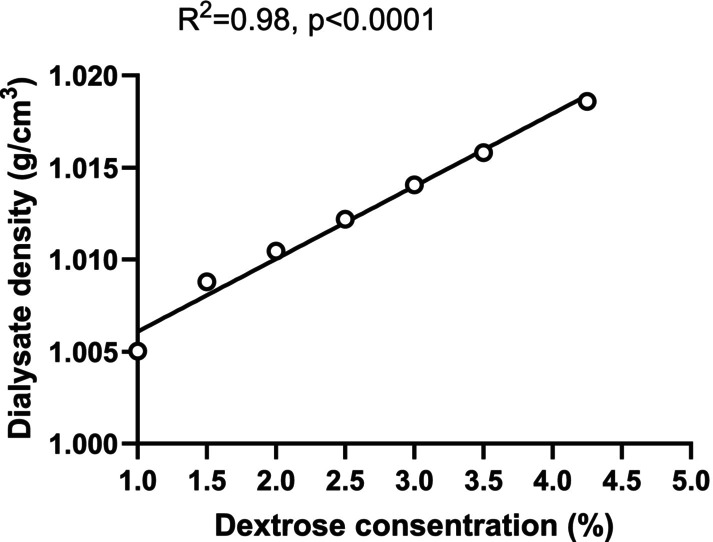
Relationship between density and glucose concentration in fresh dialysate. The samples of 1.0%, 1.5%, 2.0%, 2.5%, 3.0%, 3.5% and 4.25% dextrose were measured three times for each sample with a portable density meter.

Figure 5 illustrates a positive correlation between the dextrose concentration of fresh dialysate and the density measured by the Handheld Density Meter. These findings suggest that the density of the dialysate is influenced by the concentration of glucose molecules in the solution. Each sample at varying glucose concentrations was measured three times. Because of the consistent values obtained for each measurement, the standard deviation was effectively zero in Figure 5.

#### 4.1.1 Clinical study in PD patients

**FIGURE 6:**
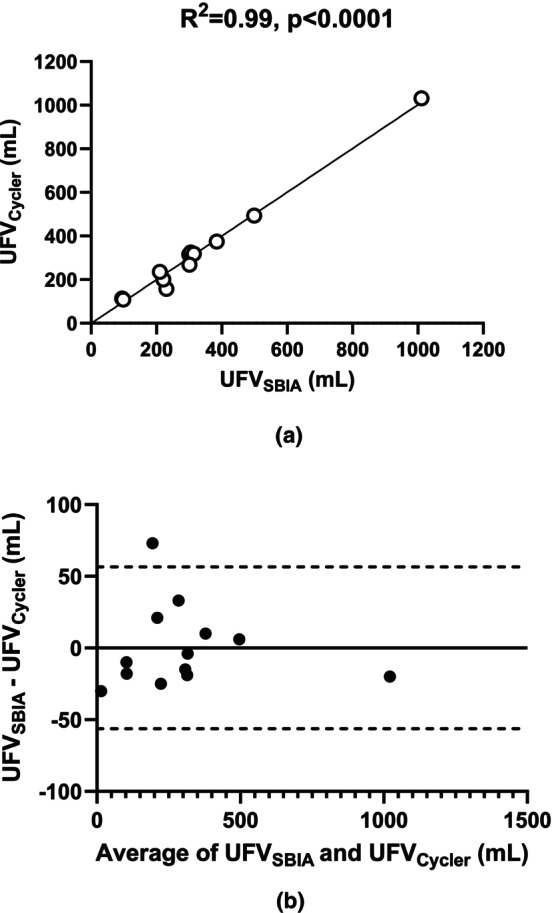
Correlation between UFV_SBIA_ and UFV_Cycler_ (a) and Bland–Altman analysis (b) in all patients (*n* = 13).

**FIGURE 7:**
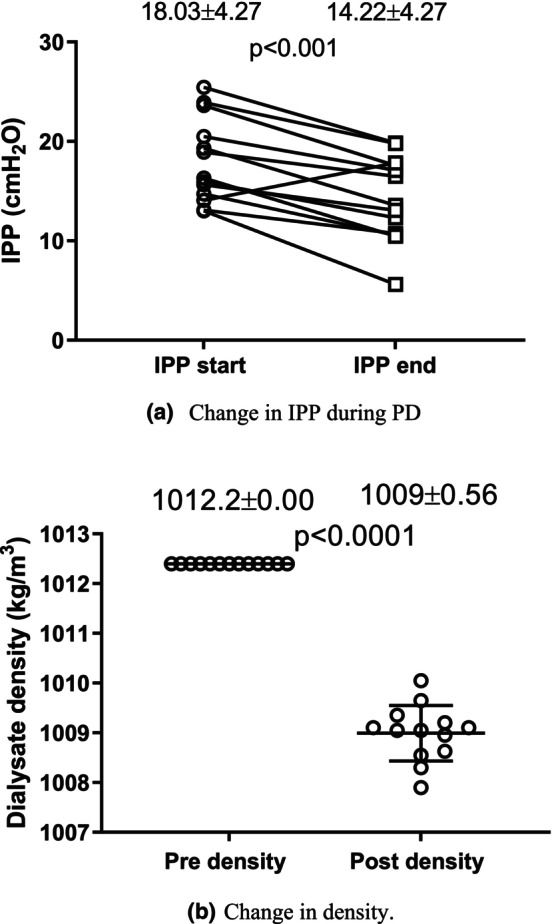
Change in IPP (a) and dialysate density (b) during PD in all patients (*n* = 13).

**FIGURE 8:**
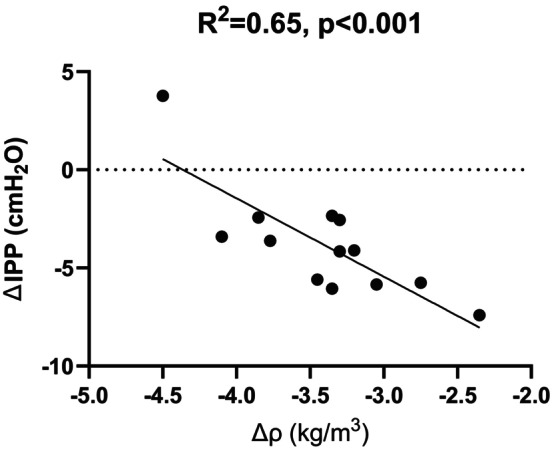
Correlation of change in dialysate density to change in IPP in all patients (*n* = 13).

**FIGURE 9:**
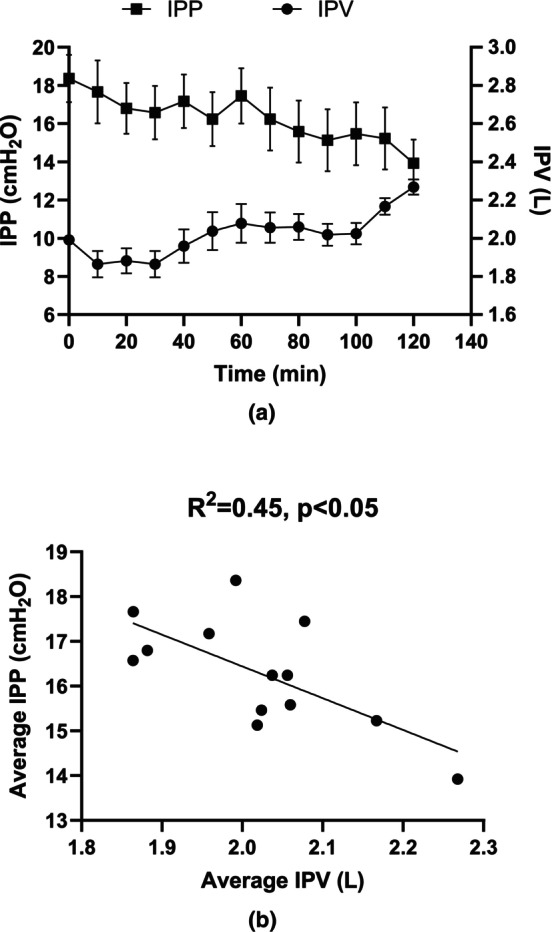
(a) shows the average values (Mean ± SEM) of changes in IPP (represented by squares) and IPV (represented by circles) across 12 patients during the dwell time. (b) correlation between average IPP and IPV from the data shown in (a). Please note that each dot in (a) and (b) represents the change in IPP and IPV during intermittent 10‐min intervals, averaged across 12 patients.

Thirteen PD patients were included in the study, resulting in a total of 15 measurements. Two patients required repeated measurements due to technical issues encountered during the initial IPP measurement. The average ultrafiltration volume was 305.1 ± 251 mL according to UFV_Cycler_ and the average UFV_SBIA_ estimated by SBIA was 305.2 ± 249 mL across all patients (*n* = 13). UFV_SBIA_ is highly correlated with UFV_Cycler_ (*R* ^2^ = 0.99, *p* < 0.0001, Figure 6a) and Bland–Atman analysis shows a bias of 0.15 ± 28.2 mL (Figure 6b). In this study, we found a positive correlation between patients' body mass index (BMI) and intraperitoneal pressure at the start of the dwell period (IPPStart), with an *R* ^2^ = 0.44 (*p* < 0.01). To explore the impact of UFV on various parameters, patients were divided into two groups based on the median UFV_Cycler_ value of 268 mL. One group had UFV_Cycler_ values ≤268 mL, and the other had values >268 mL. A summary of patient information for each group is provided in Table 1. No significant differences were observed between the two groups of patients. Figure 7 illustrates that: (a) IPP significantly decreased (*p* < 0.0001) from IPP_Start_ to IPP_End_ during the 2‐h dwell in 12 out of 13 patients. (b) The density of the dialysate significantly decreased (*p* < 0.0001) from pre‐ to postdialysate samples in all patients. This study identified a correlation between ΔIPP and Δ*ρ* (*R* ^2^ = 0.65, *p* < 0.001) (Figure 8). For those with ∆IPP <0 (#1 to #12), the average IPP decreased at 10‐min intervals (Figure 9a), while the IPV increased during the same dwell time period (Figure 9a). The correlation between the average IPP and average IPV in 12 patients is shown in Figure 9b.

**TABLE 1:** Summary of patients with different UFV.

	UFV_Cycler_ ≤ 268 mL	UFV_Cycler_ > 268 mL	*p* Value
Number of patients	7	6	
Sex	4 males, 3 females	4 males, 2 females	
Age (years)	63.0 ± 10.4	57.7 ± 16.1	0.52
Pre Weight (kg)	78.6 ± 20.5	86.4 ± 30.6	0.63
Height (cm)	170.1 ± 8.8	168.2 ± 7.6	0.7
BMI (kg/m^2^)	27.0 ± 6.2	30.0 ± 7.9	0.5
PD vintage (months)	12.4 ± 6.6	13.5 ± 6.9	0.8
IPP start (cmH_2_O)	16.9 ± 2.5	19.3 ± 5.1	0.33
IPP end (cmH_2_O)	13.1 ± 2.6	15.5 ± 5.1	0.34
ΔIPP (cmH_2_O)	−3.79 ± 1.41	−3.84 ± 3.62	0.98
Δ*ρ* (kg/m^3^)	−3.39 ± 0.42	−3.44 ± 0.64	0.88
D_2_/D_0_ Glucose	0.51 ± 0.05	0.51 ± 0.06	0.89
D/P CREAT	0.52 ± 0.11	0.50 ± 0.08	0.72

*Note*: BMI represents body mass index; IPP_Start_ and IPP_End_ represent intraperitoneal pressure at the start and at end of dwell respectively. ΔIPP represents difference between IPP_End_ and IPP_Start_. Δ*ρ* represents change dialysate density (post–pre); D_2_/D_0_ is ratio of glucose at the 2‐h dwell to glucose at start dwell. D/P is ratio of creatinine in dialysate post PD to plasma pre PD.

**FIGURE 10:**
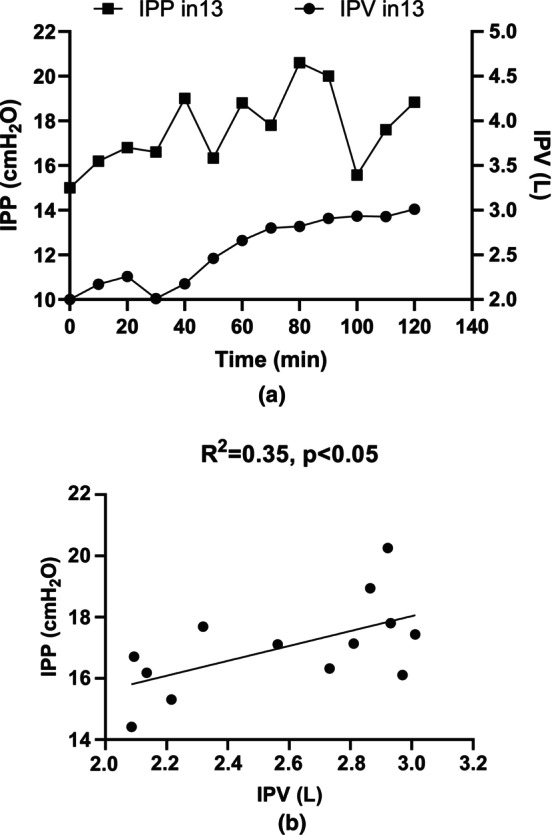
(a) Change in IPP and IPV in #13 patients in 10 min interval during dwell. (b) presents relationship between IPP and IPV with 10 min interval during dwell in patient #13 (UFV >1 L). Please note that in (b) each dot represents the change in IPP and IPV during intermittent 10 min intervals for the same patient (#13).

Although a decrease in IPP was observed alongside an increase in IPV in twelve patients (#1 to #12) during the dwell (Figure 9), an inverse trend was noted in patient #13, where both IPP and IPV increased during dwell (Figure 10a) and showed a positive correlation between IPP and IPV (Figure 10b). In Figure 10b, each point represents the relationship between IPP and IPV at 10 min intervals during the dwell period for patient #13. This study found that relative changes in ΔIPP, Δ*h* and Δ*ρ* during dwell were − 21.2 ± 18%, −20.0 ± 19% and − 0.34 ± 0.06%, respectively, while the cross‐sectional area increased from 113.4 ± 25 cm^2^ to 175.1 ± 77 cm^2^ over the course of the dwell (Table 2).

**TABLE 2:** Change in height (Δ*h*) and density (Δ*ρ*) of dialysate in individual patients.

ID	UFV_Cycler_ mL	IPP_Start_ cmH_2_O	IPP_End_ cmH_2_O	*h*_1_ m	*h*_2_ m	Δ*h*	Δ*ρ* kg/m^3^	Δ IPP cmH_2_O	*h*_1_*Δ*ρ* kg/m^2^	*ρ*_1_*Δ*h* kg/m^2^	*ρ*_1_*Δ*h*‐*h*_1_*Δ*ρ* kg/m^2^	*A*_1_ cm^2^	*A*_2_ cm^2^	Δ*A* cm^2^
#1	157	18.9	16.5	0.19	0.17	−0.02	−3.85	−2.43	−0.75	−24.65	−23.90	102.88	126.84	23.96
#2	315	15.9	12.3	0.16	0.13	−0.04	−3.77	−3.62	−0.62	−37.15	−36.53	122.13	182.19	60.06
#3	324	25.4	19.8	0.26	0.20	−0.06	−3.45	−5.59	−0.90	−57.50	−56.60	76.53	113.62	37.09
#4	318	23.9	19.8	0.25	0.20	−0.04	−3.20	−4.11	−0.79	−42.12	−41.34	81.40	113.58	32.18
#5	30	14.7	10.6	0.15	0.11	−0.04	−3.30	−4.15	−0.50	−42.82	−42.33	132.29	186.43	54.15
#6	374	13.0	5.6	0.13	0.06	−0.08	−2.35	−7.41	−0.31	−76.93	−76.61	149.42	410.25	260.83
#7	113	13.1	10.7	0.13	0.11	−0.02	−3.35	−2.35	−0.45	−24.04	−23.59	148.70	190.79	42.08
#8	268	19.3	13.6	0.20	0.14	−0.06	−2.75	−5.76	−0.55	−59.57	−59.03	100.73	162.33	61.61
#9	493	23.6	17.6	0.24	0.18	−0.06	−3.35	−6.06	−0.81	−62.41	−61.60	82.43	137.75	55.32
#10	200	20.5	17.1	0.21	0.18	−0.03	−4.10	−3.40	−0.86	−34.71	−33.84	95.00	124.82	29.82
#11	108	15.6	13.1	0.16	0.13	−0.03	−3.30	−2.56	−0.53	−26.17	−25.64	124.52	156.41	31.89
#12	235	16.3	10.5	0.17	0.11	−0.06	−3.05	−5.84	−0.51	−60.49	−59.98	119.25	207.00	87.75
#13	1031	14.1	17.8	0.14	0.18	0.04	−4.50	3.77	−0.65	40.09	40.74	138.41	164.64	26.23

*Note*: IPP_End_ and IPP_Start_ represent the IPP measured at the end and start dwell. ΔIPP represents difference between IPP_End_ and IPP_Start_ during dwell. *h*_1_, *h*_2_ represent the apparent height of dialysate at the start and end of dwell with Equation 4 respectively. Δ*h* was calculated by difference between *h*_2_ and *h*_1_; Δ*ρ* represents difference between post (*ρ*_2_) and pre (*ρ*_1_) dialysate density; *A*_1_, *A*_2_ represent the apparent cross‐sectional area of dialysate at the start and end of dwell with Equation 9 respectively. Δ*A* was calculated by difference between *A*_2_ and *A*_1_.

### 4.2 Laboratory chemical data

Table 3 shows changes in glucose, BUN, creatinine, Na+, K+, Calcium and Urea in both serum and dialysate during the PD treatment. BUN and K+ significantly decreased from pre and post serum measurement. Glucose and calcium concentration decreased significantly in dialysate, while Na + and K+ increased in dialysate during the PD treatment. It is important to note that creatinine and urea concentrations remained higher in the blood than in the dialysate after the treatment.

**TABLE 3:** Results of chemical measurements.

	Glucose (mg/dL)	Blood urea nitrogen (mg/dL)	Creatinine (mg/dL)	Na^+^ (mmol/L)	K^+^ (mmol/L)	Calcium (mg/dL)	Urea (mg/dL)
Pre Serum	114.8 ± 50.1	53.9 ± 11.8	11.4 ± 4.2	144.3 ± 6.9	4.2 ± 0.4	9.0 ± 0.7	113.9 ± 25.2
Post Serum	115.6 ± 52.7^a^	53.1 ± 11.6^a^	11.4 ± 4.2^a^	143.2 ± 6.0^a^	4.1 ± 0.4^a^	8.9 ± 0.8^a^	111.5 ± 23.1^a^
Pre Dialysate^b^	2299 ± 98	ND^c^	ND^c^	117.5 ± 2.6	ND^c^	4.9 ± 0.6	ND^c^
Post Dialysate	1170 ± 116**	38.3 ± 6.9	5.6 ± 1.8	120.4 ± 4.5	2.5 ± 0.3**	4.6 ± 0.5	80.2 ± 14

*Note*: ** represent significant differences in serum and dialysate between pre and post PD with *p* < 0.05 and *p* < 0.01 respectively.

^a^ Represents the difference between post serum and post dialysate.

^b^ Pre dialysate sample was measured from fresh dialysate bag.

^c^ ND, not detected.

### 4.3 IPP and lab data

**FIGURE 11:**
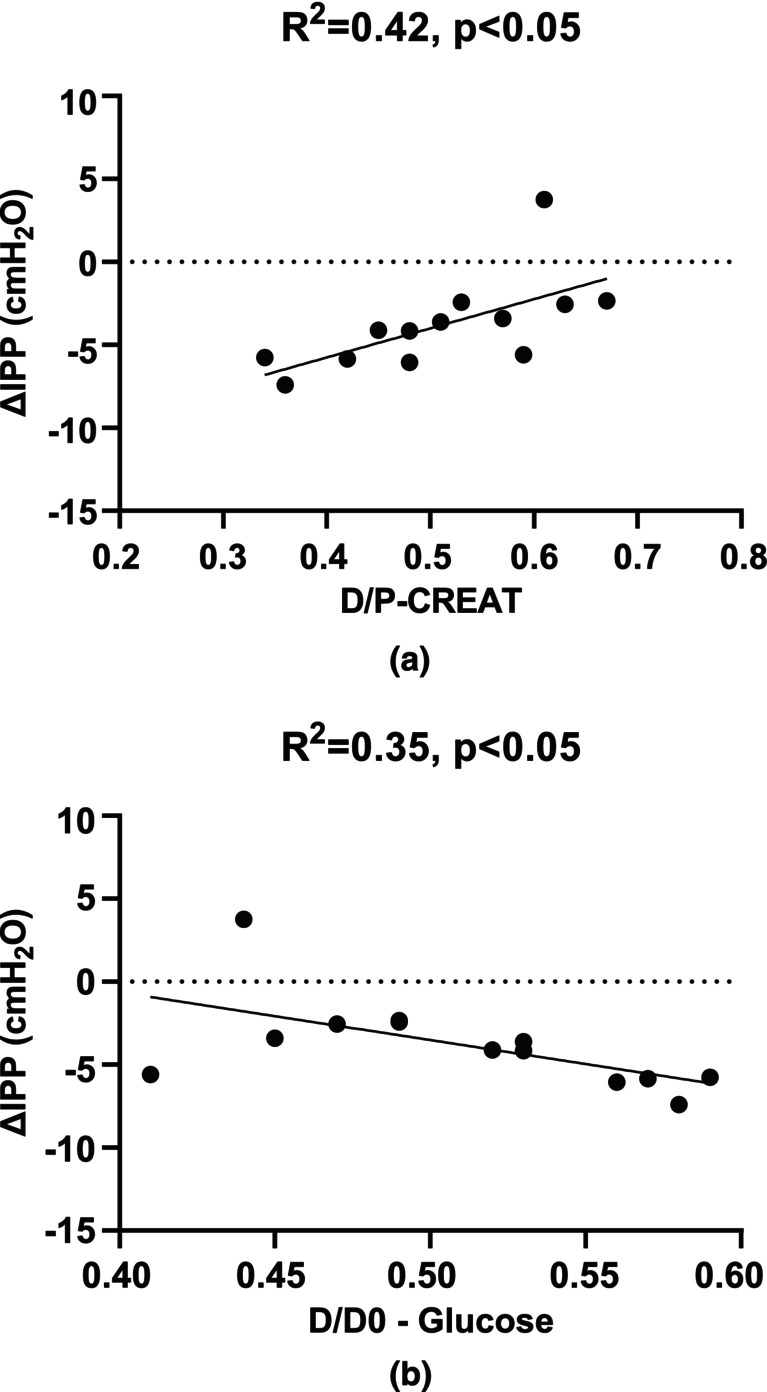
(a) Change in IPP correlated with D/P creatinine and (b) with D/D_0_ glucose in 2 h dwell time.

Figure 11 illustrates that ΔIPP was weakly correlated with the ratio of pre dialysate sample to post serum (D/P) in creatinine (Figure 11a), as well as with the ratio (D/D_0_) of glucose (Figure 11b). Given that D/P creatinine and D/D_0_ glucose ratios are commonly used to evaluate peritoneal membrane function, the relationship of ΔIPP with these transport characteristics in PD patients warrants further investigation. It is important to note that in this study, the dwell time was only 2 h, which does not meet the four‐hour duration typically required by the standard PET.

**FIGURE 12:**
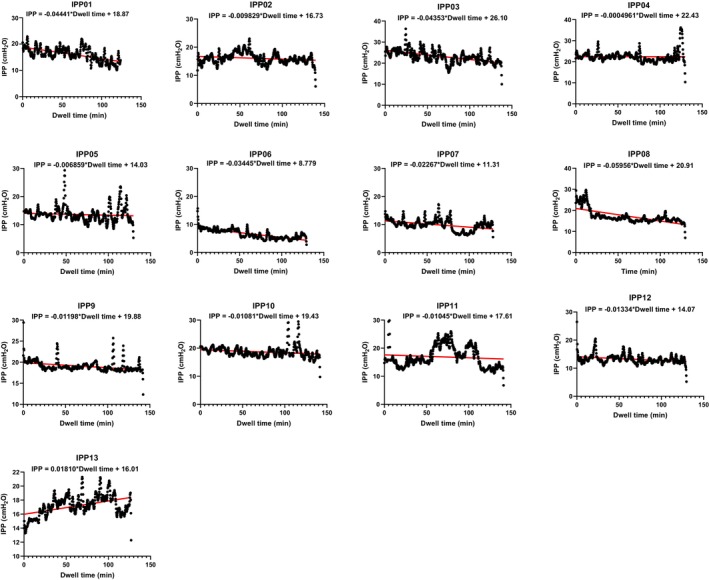
Shows all IPP data (*n* = 13) for individual patients. Regression lines (red line) and the equations were made by linear regression analysis. The results indicate negative slope in #1–#12 and positive slope in #13.

Figure 12 presents the IPP profiles during dwell in all patients. The red line represents the linear regression line for each profile. The equation (IPP = k*Time + c) on the top in each graph provides a regression formula for each patient, where k represents slope, and c represents intercept of the IPP curve. Overall, patients #1 to #12 showed that IPP decreased (*k* < 0) during dwell. In contrast, patient #13 displayed an increase in IPP (*k* > 0).

## 5 DISCUSSION

In this pilot study, we proposed a straightforward physical model to illustrate the relationship between IPP and IPV in the peritoneal cavity during PD dwell. The model demonstrates that IPV acts as the driving force for changes in IPP, primarily depending on alterations in both the height and the cross‐sectional area of fluid in the peritoneal cavity. The bench study demonstrated that the sensor integrated into the automated PD cycler provides accurate and precise measurement of IPP. The key findings are as follows: (1) A threshold determines whether IPP increases or decreases during dwell, (2) The reduction of IPP as IPV increased during the dwell is attributed to an increase in the cross‐sectional area (ΔA) of IPV in the peritoneal cavity, (3) The increase in IPP during dwell results from an increase in the height of the dialysate solution in the peritoneal cavity. Furthermore, the study found that ΔIPP was associated with the characteristics of peritoneal membrane transport: D/D_0_ of glucose and D/P of creatinine. This suggests that ΔIPP may serve as a valuable, additional reference in clinical practice for evaluating the transport characteristics in individual patients.

Recently, IPP has emerged as an important focus of research in clinical PD studies (Dejardin et al., 2007; Ferreira, 2023; Giuliani et al., 2020; Outerelo et al., 2014; Perez Diaz et al., 2017; Sigogne et al., 2020; Twardowski et al., 1983). Understanding the dynamics of IPP and its relationship with various parameters has the potential to enhance our comprehension of PD therapy and improve patient outcomes. This knowledge may shed light on the underlying mechanisms driving these applications and their clinical improvements.

Previous publications have reported a positive correlation between IPP and an increase in IPV when dialysate is filled into the peritoneal cavity (Dejardin et al., 2007; Twardowski et al., 1983, 1986). However, others have found that IPP decreased when IPV increased (as indicated by UFV) during dwell (Durand et al., 1993; Perez‐Diaz et al., 2021). In the study we confirm previous findings (Durand et al., 1992), demonstrating an inverse correlation between ΔIPP and UFV_Cycler_ (*R* ^2^ = 0.47, *p* < 0.05) in twelve patients (#1 to #12) who experienced a decrease in IPP during dwell (Figure 8a). This discrepancy highlights the complexity of IPP dynamics, suggesting that additional factors may influence IPP during the dwell period. Further research is needed to elucidate the mechanisms underlying these observations (Durand et al., 1992; Perez‐Diaz et al., 2021). Investigating potential factors contributing to these contradictory findings could provide valuable insights into the complex interplay between IPP and IPV during PD (Perez Diaz et al., 2017).

Our model provides a valuable framework for illustrating the dynamics of fluid movement during dwell in PD. It demonstrates that during dwell, excess fluids from surrounding tissues and internal organs are removed into the peritoneal cavity via ultrafiltration. This process not only increases IPV but also expands the cross‐sectional area of IPV within the peritoneal cavity. Understanding these mechanisms is essential for comprehensively elucidating the relationship between IPP and IPV during peritoneal dialysis. In this study, the observed increase in cross‐sectional area from 110.4 ± 24 cm^2^ to 155.5 ± 32 cm^2^ accompanied by a decrease in IPP during dwell suggests that IPP decreases as IPV increases up to the certain threshold level which determined by individual maximal expandable across‐sectional area. If IPV continuously increases and beyond this threshold, IPP will rise. This linear correlation between IPP and IPV is attributed to a constant cross‐sectional area and an increase in the height of IPV in the peritoneal cavity, which helps to explain the relationship between IPP and IPV during the filling and draining phases.

To quantitatively analyze IPP data, we developed the equations (Equation 10) to identify the threshold for the relationship between IPP and IPV during the dwell period in peritoneal dialysis. Due to the variability of the peritoneal membrane among individual PD patients, there is a threshold that determines whether IPP increases or decreases during dwell. It depends on a difference between change in IPV (UFV) and change in cross‐sectional area (*h*_1_*Δ*A*) times initial height in the peritoneal cavity. As UFV increases during dwell, the cross‐sectional area of the peritoneal cavity expands, which in turn decreases the apparent height of the dialysate (Δh), leading to a decrease in IPP. As a result, IPP decreases. However, if UFV continues to increase after the cross‐sectional area reached its maximal value, the height of fluid in the peritoneal cavity will rise, eventually, causing an increase in IPP. In our study only one patient (#13) exhibited ΔIPP>0 (Figure 10). This was attributed to a significant ultrafiltration volume (UFV) exceeding 1 L, which increased the apparent height (Δ*h*) of the dialysate by 0.04 m.

This threshold may additionally be influenced by the elastic properties of the peritoneal membrane. Due to these properties, a small increase in fluid volume within the peritoneal cavity may not exhibit a linear correlation with the height of the fluid as the cross‐sectional area expands. This nonlinear relationship arises because the membrane can stretch and expand up to a certain extent, altering the cross‐sectional area and affecting fluid height in a complex manner. Consequently, the increase in diameter enlarges the cross‐sectional area, resulting in a decrease in pressure. This provides an additional explanation for the inverse relationship between changes in IPP and UFV. Additionally, body composition, such as body mass index (BMI) may influence the IPP measurement, as higher BMI could reduce the body elastic properties (Sigogne et al., 2020). Given the variability in individual body geometry, weight, and peritoneal cavity size, it is difficult to establish a reliable formula for estimating IPP based on anthropometric measurement and BMI (Li et al., 2023; Sigogne et al., 2020).

Based on the principle described above, if the threshold <0, ΔIPP will show an inverse correlation with UFV during the dwell period. Figure 9b demonstrated a relationship similar to that observed in previous publications (Durand et al., 1993), confirming the findings of this study (*n* = 12). As mentioned earlier, this inverse correlation is due to multiple factors, including an increase in UFV, a decrease in dialysate density, and an expansion of the cross‐sectional area of peritoneal cavity. On the other hand, if threshold >0, IPP will show a positive correlation to the increase of IPV (Figure 10b). This is primarily attributed to a significant increase in fluid volume within the peritoneal cavity, resulting in a linear increase in height. This observation aligns with findings from previous publications (Dejardin et al., 2007; Twardowski et al., 1986). In this study, we found that, unlike the filling and draining phases of PD treatment, the relationship between IPP and IPV during the dwell phase is complex and depends on two major factors: (1) Increase in the height of dialysate in the peritoneal cavity will increase IPP; (2) Expansion of the across‐sectional area of the cavity can lead to IPP decline. In general, with our model, an IPV threshold will determine whether IPP increases or decreases. The change in IPV during the dwell phase relies on a combination of ultrafiltration and absorption by the individual peritoneal membrane.

This study demonstrates only weak correlations between ΔIPP and both the D/P creatinine and D/D_0_ glucose ratios measured after a two‐hour dwell (Figure 11). In clinical practice, the four‐hour D/P creatinine and D/D_0_ glucose ratios are standard measurement for evaluating membrane transport characteristics in PD patients. Our findings provide valuable insights into the relationship between ΔIPP and peritoneal membrane function. Individual differences in the elasticity and transport characteristics of the peritoneal membrane may contribute to IPP variations. In addition, the measurement of IPP may be influenced by other physiological signals, such as breathing and intestinal movements. Moreover, the dwell time in this study was 2 h, which is half of the standard dwell time (4 h). All these factors could explain the weak correlation between ∆IPP and transport characteristics (Figure 10). Although our study utilized a two‐hour dwell time, the results suggest that ΔIPP could be a clinically relevant parameter for categorizing membrane transport characteristics in the future. However, further testing with a four‐hour dwell time is needed to confirm this.

It is crucial to understand the factors that may affect the accuracy of IPP measurements. According to Equation 2, the relative height between the automated PD cycler and the patient's chair must remain constant, as the absolute value of IPP is determined by this height. Additionally, it is essential for pastients to maintain a consistent body position during IPP measurements to minimize potential interference from body movement. Furthermore, certain physiological factors, such as breathing and intestinal motion, can introduce noise in the analysis of IPP. Therefore, employing a high‐quality filter with signal processing is imperative to obtain reliable IPP data.

While this study confirmed that IPV can be measured using SBIA, the method requires specialized equipment and trained operators to ensure accuracy, making it impractical for home use by the PD patient. In contrast, IPP monitoring holds promise for use in PD patients. It is important to note that IPP is currently measured using a manual and intermittent method, whereas our technique offers a noninvasive, automatic, and continuous way to monitor IPP with a regular PD cycler machine. It is also crucial to distinguish IPP from peritoneal hydrostatic pressure. The latter refers to the pressure between blood (capillary) and interstitial tissue (Wiig et al., 1986; Zakaria et al., 2000). IPP in this study refers to the pressure produced by the height and composition of fluid in the peritoneal cavity due to gravity. A major weakness of this pilot study is the small sample size, and large trials are needed to confirm potential clinical implications identified in this research.

## 6 CONCLUSION

In this study we presented a noninvasive and simple method for continuously monitoring IPP using an integrated sensor within the automated PD cycler, while IPV was monitored with a bioimpedance device. We proposed a physical model to explain the relationship between IPP and IPV and confirm this relationship with clinical data. Change in IPP primarily depends on two factors: the height and change in the cross‐sectional area of fluid in the peritoneal cavity during dwell period, which are driven by IPV. Our finding indicates that ΔIPP is associated with established clinical markers of membrane transport characteristics in individual PD patients. Measurement of IPP could provide useful information about the dynamics of fluid change in the peritoneal cavity.

## AUTHOR CONTRIBUTIONS

FZ, LR, LT, JY, MV and RL performed clinical and bench experiments; ZH, XW and NG performed chemical measurement for the dialysate and serum samples; FZ, LR, KP, KF, PC, BS and PK designed the study protocol; FZ analyzed data and drafted manuscript; FZ, XW, LR, NG, RL, BS and PK revised manuscript.

## FUNDING INFORMATION

This project was funded by the Renal Research Institute.

## CONFLICT OF INTEREST STATEMENT

The Renal Research Institute is a wholly—owned subsidiary of Fresenius Medical Care North America. P.K. holds stock in Fresenius Medical Care. FZ, LR, LT, ZH, XW, NG, PK are Renal Research Institute employee and JY, KF, KP, RL, PC and BS are Fresenius Medical Care employee.

## ETHICS STATEMENT

The study was approved from the Western Institutional Review Board (WIRB Protocol #20202653), and was performed according to the ethical principles of the Declaration of Helsinki.

## Data Availability

The data for supporting the findings of the study are available from the corresponding author upon reasonable request.
